# Observations on Cerebro-Arachnitis, or Miningitis

**Published:** 1851-11

**Authors:** James Smick

**Affiliations:** Indian Point, Menard Co. Ill.


THE NORTH-WESTERN
MEDICAL AND SURGICAL JOURNAL.

VOL. IV.] NOVEMBER, 1851. [No. 4.

### Part I.—Original Communications.

ARTICLE I.

*Observations on Cerebro-Arachnitis, or Meningitis, as it occurred in Clark and also Menard Counties, Ill. Read before the Central Medical Society of Illinois by* JAMES SMICK, M. D.

No person that has ever had cases of Meningitis, in a malignant form to treat, will lightly esteem any information relative to the Ætiology, pathology and treatment of it, from any source however humble.

Having seen many cases of this fatal disease, in the course of 17 years' practice in Darwin, Clark County, and some since I came to Menard, I propose giving to the society the results of my experience relative to it; and if it does nothing more than call forth the observations of abler writers, on the subject, I shall be well paid for my trouble.

The first cases that I saw, were in 1832, and I have seen more or less of it every year since. In 1841, and also in 1845-6, Meningitis raged as an epidemic, in Darwin and vicinity in Clark County, Ill. It was much more fatal in these years, than when only a few sporadical cases occurred, owing to the increased malignancy attending it.

The epidemic did not rage to the same extent in 1841 and 1845 that it did in 1846. In the winter of 1845 and '46 the malignant Erysipilas or "Black Tongue," as it is termed, made its appearance. It commenced some time in December, and raged until warm weather set in, in the spring.—About the middle of March, the first cases of Cerebro-Arachnitis made their appearance, and continued to occur until the middle of June following. The period of its greatest prevalence was in April, this year. The disease was less tractable to medicines in this month, than any other. In 1841 we also had more cases in the month of April. In 1845 the first cases occurred the last of September, and the last about the middle of November following; no more then until it commenced in March. I had cases in almost every month of the year, but none from September until May; fewer in June, July and August, than any other months of the year.

The few cases that came under my observation since I came to Menard, occurred between the months of October and May. Two cases in 1848, seven in 1849, and one in 1850. No disease that has fallen to my lot to treat, has been so fatal as this one. Asiatic Cholera cannot boast of a greater mortality. It is not unusual for one half or more of the cases, to terminate fatally. By reference to the Medico Chircergical Review, No. 86 for 1841, we see that the disease prevailed at Versailles, Rockport, Metz and Strasbourg. The history of the epidemic as it appeared at Strasbourg, is given by Dr. Wunchendorff. The mortality is even greater at these places than we have stated above. "One half," says the Dr. "of my patients died; but this mortality is common with this epidemic, with sporadic Meningitis, which usually seems fatal in two out of every three cases. In the Civil Hospital at Strasbourg, 21 patients died out of 40 that were admitted; and in the Military Hospital the mortality was still greater—104 out of 176." A small fraction over a third of the cases in my practice proved fatal. Of the 10 cases that I had since I came to Menard, four proved fatal—two were in "articulo mortis" when I saw them.

*Ætiology.* It may be presumption in me to attempt to trace out the cause of epidemic Meningitis, as all who have written upon the subject, so far as I know, have given so few theories, or hypothesis to build upon. But I wish to state facts, so far as I have observed them, and draw from them, such deductions as they may justify, in order that the true cause may be discovered.

The fall and winter preceding the epidemic, that we had in the spring of 1841, were unusually wet. The Wabash river was very high, overflowing its banks many weeks together, as well as the small streams that empty into it near Darwin; leaving large heaps of earth and silt washed together, by the water, when it subsided in the spring. The same was the case before the epidemic of 1845. All remember the high water of 1844, which did not subside, very low until 1845. The fall of this year was dry; but in December it commenced raining, and raised the streams to high water mark. When the water subsided in the spring, more than a usual amount of silt was left on the bottoms. So far as any mention is made of the weather, by writers on this subject, say the disease was preceded by a great deal of wet weather.

This was the case with the epidemic that prevailed in Rutherford County, Tennessee, in 1842, as given by John W. Richardson, M. D., in his report to the Medical Society of the State, for that year, (vide Western Journal of Medicine and Surgery, Vol. vi. No. iv.) Also the case with the epidemic Cerebro-Arachnitis, that raged in Boone and Galoway Counties, Missouri, after the great freshets of 1844 and '45. A large amount of silt and earth was washed up and left on the bottoms after the water subsided. To this cause some of the physicians of those Counties attributed the epidemic.—Do they not stand one to another as cause and effect? If so, what is the agent or agents?

The first question here propounded, may very safely be answered in the affirmative. The second rests entirely upon conjecture ; owing to the imperfection of our science we have failed to detect any agent, to which we may attribute it.

As to the first question, I think the facts above enumerated prove that Meningitis follows freshets succeeded by warm winters. Whether " these things of themselves are sufficient to produce the epidemic influence of the atmosphere, I am not able to say ; but one following in the train of the other, I think they at least demand a close investigation. I will mention a few facts, that came immediately under my own observation, that will perhaps, throw some light on the subject.

About three fourths of a mile below Darwin on the Indiana side of the river, there was a farm with some 15 or 20 acres of ground cleared, that had been cultivated some two or three years ; a small cabin stood upon it. In 1844 the family that lived on this place had to leave in consequence of high water that occurred in the month of June (it always overflowed in a high stage of water,). The water kept up for some time, so that no one attempted to occupy it till the spring of 1846, in the last of March and first of April. The man who expected to occupy it engaged some friends to repair the cabin and assist in putting up the fence, which was thrown down by the high water. The company consisted in all of about fifteen men, women and boys. They made what is usually termed here "a frolic" to repair the farm. The women went along to cook for the others. Whisky was freely used on the occasion. It took them two days to accomplish the work. Ten out of the fifteen took Cerebro-Arachnitis ; seven of whom died. The period of incubation was from three to ten days. The first case occurred the third day after they quit work, and the last the tenth. Two of the first cases were fatal ; the first one only lived about six or eight hours from the first feelings of indisposition. The last cases were not so severe as the first ; most of whom recovered.

I think drinking on the occasion was not the cause of the disease, for the following reasons:

1. The intemperance on this occasion was no greater than a portion of them were in the habit of indulging in every day.

2. Those who did not use spirits were also affected.

3. The "frolics" which were made on the prairie, were not followed by the same results.

That the cause of the disease existed on the premises, I think there can be no doubt, from the fact that so many were taken sick out of those that engaged in the work; and that they lived some five or six miles apart, belonged to four families, and were taken sick so nearly together; while the members of the families who did not work on this place escaped the disease.

The amount of silt left on the field by the water and drifts of wood out of which the rails were taken, was well calculated to send forth what is usually termed "*malaria*," if it had been at a season of the year when the temperature was sufficiently high to produce it. May not malaria produced by a high temperature be the cause of Bilious fever, and malaria produced by a low temperature the cause of Cerebro-Arachnitis? The foregoing facts go to prove this.

In what this malaria consists, I am not able to say. Whether it consists of heat and moisture, or some gaseous emanations from the earth, such as carbonic acid, carbonic oxide, carburetted hydrogen, sulphuretted hydrogen, carbonate of ammonia, or cryptogamia, such as is termed the vegito-animalcular hypothesis, I cannot say. But be it what it may, it seems to be generated in the fall season of the year, that keeps the disease up through the winter, and when a sufficient expansion of caloric takes place in the spring to generate it, the disease increases. Or, as we have many cases on record of malaria laying in the system for a considerable length of time in a latent state, before it produced disease, might the malaria generated in the summer lay in the system in a latent form until cold weather brought on some exciting cause of disease that would so modify it that the attack would be Cerebro-Arachnitis, in place of Bilious Remittent Fever? I rather think not.

*Pathology.*—Relying on its effects to guide us in an estimate of its character, we may say that efficient cause of Cerebro-Arachnitis is a sedative and irritating quality, somewhat like the narcotic-irritating gases; or certain solid and fluid bodies, which, in large doses, destroy life suddenly, by reducing power, and in smaller portions weaken while they pervert the functions, producing reduction of vital energy, obtuseness of sensibility, suspended or perverted secretion and diminished calorification, and from an equal necessity they will be felt in all parts of the body, because the agent which produces them travels with the circulation.

If we suppose such matter to be simultaneously introduced into all the serous sacks of the body, we should expect immediate reduction of the vital powers and early death; though we can conceive of the quantity being so small that the system would react, and fever and inflammation ensue. And it requires no great stretch of the imagination to suppose that the inflammation was determined to the Arachnoid membrane of the brain and spinal marrow, in this case, by the same law that governs the virus in other cases, producing diseases and inflammations of their own peculiar character. Such as the virus of Small Pox, a peculiar eruption and inflammation of the surface; the virus of Scarlatina determines its action upon the skin and throat; and Erysipelas upon the skin.

May not the cause, by the same law, determine the inflammation to the Arachnoid membrane, in Cerebro-Arachnitis, in those cases where they survive the first shock?

In those cases that die suddenly—in which no reaction follows the chill—the powers are so reduced that they die as individuals die under the influence of prussic acid, or some other poison of a like kind. Their susceptibilities to the various sustainers of life are annihilated, and they sink. Although the surface is cold they do not shiver nor complain of cold, because the functions of their nervous systems are too deeply smitten to admit of their action on the muscles, or of their taking cognizance of the loss of caloric. And the more complete this symptom, the less hope we can have of reaction taking place. But if death does not follow the first impress of the poison, according to a physiological law that exists, that, after *depression* there will be *elevation*, the vital forces arise from their state of depression, and excitement is the result.

In this stage of the disease the inflammation seats on the Arachnoid membrane, and also, in some cases, the substance of the brain itself constituting what is called by Rollet Cerebro-Spinal Meningitis, and Encephaloid Meningitis, according as the envelops alone of the cerebral centres, or the cerebral substance itself participates in the inflammation.

Post mortem examinations exhibit active congestion, suppuration, and even softening of the brain and spinal marrow, according to the degree and intensity of the disease.

Dr. F. M. PAYNE, and myself, made a post mortem examination of a boy that died from this disease, in December, 1842. He died the 6th day from the attack. The last 24 hours before he died he had some four or five hard convulsions. The examination was made some twelve hours after death. On opening the cranium the external blood vessels were highly congested; the Arachnoid membrane was inflamed in every part of it, and covered with a thick layer of pus. This extended as far down the spine as we were able to see. The corticle substance of the brain seemed to be slightly softened, the medullary portion was normal, there was rather more water in the ventricles than in the healthy state. We examined no other parts of the system, as the symptoms did not indicate any particular derangement of any other part; and the amount of disease that we discovered in the cranium we thought was sufficient to account for the death of the patient.

I have seen in a good many cases an Erysipelatious eruption, follow the inflammation of the Arachnoid membrane. This eruption commences about the time the inflammation leaves the brain. Its general location seems to be the throat under the chin, and the face. Case 3d is an example of this kind.

Can the inflammation of the Arachnoid membrane be exclusively of the erysipelatious character? If so, this affords another reason why it seats upon the surface of the brain and Arachnoid membrane. Death is evidently produced in those cases that die in a few hours from the attack, by congestion of the brain and enervation, or rather paralysis of the nervous centres; and those that reaction takes place in, die from the result of inflammation of the Arachnoid membrane and brain.

*Symptoms.*—This disease has its exacerbations and remissions, very much like Bilious Remittent Fever, or most of the diseases of a miasmatic origin.

The type is mostly quotidian, and in some cases it is tertian. Each exacerbation, in some cases, is preceded by a chill, or chilliness ; and the more distinct the chill, the exacerbation and remission, the mere manageable the disease.

The fever, in some cases, is of a Synochus, and in others, of the Typhoid character.

The disease is usually ushered in with a chill, which lasts for three or four hours, and in some cases the coldness does not amount to a chill, only a chilliness. This is soon followed by a reaction that runs high in many cases; in others the reaction is feeble and broken. These are the bad cases, if attended with other symptoms denoting malignancy. About the first appearance of reaction, the patient complains of a severe pain in his head and back of the neck; in some cases the pain commences suddenly as though he was struck with a hammer, and continues of a throbbing kind, which is very annoying to the patient. Some cases are attended with a severe pain in the back and loins. Sickness and vomiting frequently attend. When the reaction runs high, and in those cases attended with malignancy, they become restless, talkative, boisterous, calling those to whom they were most attached in health, pulling and scratching the bed clothes, sometimes biting their finger nails, and occasionally screaming as though they were frightened. Others lay perfectly stupid, muttering incoherently, and when aroused would answer questions rationally if addressed in a sharp, quick tone, and then would immediately commence writhing and twisting, and talking incoherently again. Those who die before any reaction is established, roll from one side to the other, and toss about in every possible manner, apparently insensible to all surrounding objects; and if aroused at all, are not sensible of their danger or even that anything is the matter with them. And those who die by congestion of the brain, the breathing becomes stertorous before death. In most cases a free perspiration sets in, soon after reaction is established, which continues through the exacerbation. Deafness attends in most cases. Obscure vision, double vision, and even blindness, attends in many cases. The head is drawn back between the shoulders, and the whole spinal muscles contracted so as to form the body in a curve backward, in nearly all the bad cases, which incapacitates the patient from laying comfortably upon the back, such as exists in that form of tetanus called Opisthotonos. The pulse in the cases attended with the sinking of the vital energies of the system, is feeble and not very quick; during the exacerbation it is quick and frequent, beating mostly over 100 and sometimes 150 beats in a minute; and in some cases it is hard. The tongue at the commencement is not much affected, but after a few paroxysms it becomes white, sometimes yellow, and in the cases where the fever assumes a Typhoid character, it is covered with a thick brown or black coat, which becomes very dry; the tongue, in this case, cracks. The bowels, in the most of cases, are torpid; all the secretions seem to be arrested or retained. The urine is not discharged for some hours, and is mostly of a high color.

Sometimes the arms, and at others one or both legs, are paralyzed. An eruption frequently makes its appearance on the surface, which is confined to the forehead, breast and arms; it is in the form of red specs. In some cases the throat is sore, and in a few I have seen paralysis of the muscles engaged in deglution exist, so that it was almost impossible to swallow. It is not unfrequent—if the cases are protracted—for severe spasms or convulsions to attend; this is a very unfavorable symptom.

Delirium is a prominent symptom of this disease, but in some cases the mind is not affected, the patient retains his senses through its whole course.

*Prognosis.*—Cerebro-Arachnitis has no certain number of days in which to run its course; and is not marked by any critical days. The first cases that occur in a place, when it assumes an epidemical form, are much more malignant than after it has existed in this character for a while. Sporadic cases yield more readily to medicines than when it rages as an epidemic. This is the case so far as I have observed. The more protracted the chill, the more broken the reaction, and the greater the nervous prostration, the greater the danger; especially if the sensorium is deeply affected at the same time,—that is, if the surface is cold and the patient is not sensible of this absence of caloric, delirium, restlessness, &c., while the more open the excitement, the longer the remission and the more sensible the patient is of all his aches and pains, the more favorable the result.

A great many cases die in the first chill; and if they survive this, and the reaction is broken, they sometimes die in the second exacerbation. Convulsions, if they occur in the disease after it has existed some days, form an unfavorable smyptom, for most of the cases in which I observed it proved fatal. In most of those cases where the nervous system was deeply affected, and convalescence from the first attack was established, the recovery was very slow. In some, swelling of the joints, and phlegmonous inflammation of the muscular tissue—mostly upon the arms and legs—followed as a sequel.

*Diagnosis.*—Cerebro-Arachnitis may be distinguished from Cynanche Tonsillaris, or Cynanche Maligna, by the absence of inflammation of the tonsils in many cases, and when it does occur the swelling is not so great; and when the eruption does occur it is in more circumscribed spots, not so large as in either form of Cynanche.

It may be distinguished from Congestive Chill by the disease running a much more rapid course. The patient generally survives the first and second chill in Congestive Chill, but in Cerebro-Arachnitis when he dies in the chill it is usually the first; and the sensorium is more affected in this than in Congestive Chill.

It may be distinguished from Typhoid Fever from the rapid course the disease runs, and the prominence of the leading symptoms. And from Bilious Remittent Fever by the absence of the bilious diathesis, and the greater amount of pain in the head, and opisthotonos, when that exists. And also from Pneumonia, from the absence of the pneumonic symptoms, as pain in the region of the lungs, cough, &c.

*Treatment.*—The first indication to be fulfilled is to bring about reaction, and prevent the rapid sinking of the vital energies of the system.

The second indication is to lessen the inflammation of the brain, and moderate the excitement.

The third indication is to intercept the paroxysms, so as to arrest the disease in its course.

The remedies that have succeeded best in my hands to accomplish the first indication, are, first, sinipisms, as strong as they can be made, to the entire spinal column and abdomen, and also envelope the lower extremities with the same. These should remain on as long as the patient can bear them, if he is sensible of their effects,—if not, until they have made a strong impression. When these are removed the surface should be rubbed with a strong effusion of cayenne pepper and vinegar. The rubbing should be performed under the bed clothes, so that the air does not come in contact with the moist surface; and it should be performed diligently and perseveringly, until reaction takes place, or all hopes of its taking place are given up. Artificial heat should be applied to assist these in bringing about reaction. This may be done by applying warm stones, warm bricks, or ears of corn boiled in water, around the patient, especially to the lower extremities, until reaction takes place.

Internally I give a powder composed of the bi-chloride of mercury and pulv. camphor, of each 10 grains, and pulv. opium ¾ of a grain, every two or three hours, until reaction takes place; and after it takes place I reduce the quantity of camphor to two grains, and repeat until a decided impression is made on the bowels, or seven or eight powders are given. I also use sulph. ether and spirits ammonia, to bring about reaction, and a remedy that I think well of, but have not had much experience with in this disease, is liquid camphor, or three parts of camphor dissolved in one of chloroform; ten or twelve drops of this should be given at short intervals until reaction takes place.

The remedies that I use to fulfil the second indication are, externally, cold applications to the head, cold water or ice applied to the head—if the cold water is used, it should be done by pouring on, or applied with a sponge often enough to keep the head cool. A large blistering plaster long enough to cover the cervical vertebræ, should be applied to the back of the neck. The spinal column below the blister should be rubbed frequently with a liniment made of equal parts of hartshorn, camphor, and spirits turpentine. Rollet is in the habit of applying the actual cautery to the spinal column, with marked advantage. I have never used it, but will in the first case that requires it.

When the fever runs high and the heat of the surface is great, and also dry, it should be sponged off frequently with warm vinegar and water, or weak soap suds; this should be done often enough to keep the heat down.

Internally, I continue the calomel, camphor and opium powders used to bring on reaction, or if I have not been called until this stage of the disease, I commence them immediately and give them until free catharsis is produced, or seven or eight powders given; if they do not operate, follow them with a dose of castor oil or seidlitz powders. I give some four or five powders of the above kind, followed with a cathartic if necessary, every exacerbation, or once every twenty-four hours if the exacerbations are not well marked, until all marks of inflammation subside, or the mouth is affected by the calomel. When the pulse is hard and no symptoms exist that indicate a sinking of the vital energies of the system, I bleed from the arm until a decided impression is made upon the circulation, and repeat if necessary. There is no remedy that requires more caution, or discretion in using it, than bleeding in Cerebro-Arachnitis; for there is a strong tendency throughout the course of the disease to the sinking of the vital energies of the system, and when venesection increases this tendency, we should not use it; but where the excitement is open and the inflammation runs high, we may venture upon it without any hesitancy.

Rollet, and other French physicians, rely with much confidence on depletion and antiphlogistics in the treatment of this disease. But the same success has not attended my use of the same remedies. I therefore discriminate in the cases in which I now use them, especially depletion. In all cases marked by a remission, during the remission I use quinine, morphine, and precip. carb. ferri., to intercept the paroxysms, which is the third indication of cure.

This is the great antiperiodic remedy of the age, and all diseases of miasmatic origin marked by exacerbations and remissions, demand its use. In the first remission that presents itself I use a powder composed of from six to eight grains of quinine, ¼ grain morphine, four grains precip. carb. ferri., one every two hours during the remission, or until the system is brought under the influence of the quinine. I use this compound in this way during each remission, until the disease is broken up. If there is much stupor or somnolency I omit the morphine.

The only class of cases that I have not seen the tonics benefit is that class attended with a dry or parched tongue. Quinine has been as beneficial in this disease, in my hands—in the cases where it can be given—as it is in Bilious Remittent fever.

It may be said that the cases in which it is beneficial would recover without it. This, according to my experience, is not correct; for many recover under its influence presenting the same group of symptoms that, before I used it at all, proved fatal. And, further, the same success did not attend the practice of the physicians that did not use it, in the same epidemic, as it did those who did.

The remedies here detailed in the three grand indications of cure, are those that I rely upon, and that have proved most successful in my hands, in controling this formidable disease, Cerebro-Arachnitis. But in a disease presenting such a variety of symptoms, many symptoms may arise during its course that the remedies above given will not reach; therefore each symptom should be met by its appropriate remedy. I will give in detail three cases from the many that I have treated, as a further illustration of the disease:

*Case* 1st. I was called to see Mrs. M——, April 9th, 1846. She was taken about three hours before with what they supposed to be an ague chill. She was 18 years of age, of a sanguino-bilious temperament; the mother of one child; had always enjoyed good health. I found her tossing from one side of the bed to the other, pulling the bed clothes, talking incoherently, repeating mostly monosylables. These restless spells would last for 20 or 25 minutes; they would be succeeded by stupor or somnolency that would last about as long. The extremities were cold; the pulse was small but not very frequent, rather shattered; respirations rather more frequent than in health, the inspirations were long and heavy; appears insensible to all surrounding objects, but when spoken to in a loud sharp tone, she will seem to pay attention at the time, but would not answer questions. Said when she was first taken the pain was in her head. I ordered mustard poultices to the back and bowels, and the extremities rubbed with Cayenne pepper and vinegar, cold applications to the head; gave a powder composed of 10 grs. Sub. Murias Hyd. ½ gr. Pulv. Opii, 10 grs. Pulv. Camphor and repeat every 2 hours; also, a half teaspoonful of of Sulph. Ether to be given every half hour. I visited her in company with Dr. Seth N. Montague, (who was practicing in partnership with me at the time,) in an hour and a half from the time I left her. No change for the better. The rubbing and artificial heat that had been applied increased the heat of the surface, but it was not of that character that indicated reaction. We continued the treatment. Dr. Montague remained with her and did all that could be done, but in an hour and a half more she was a corpse.

*Case* 2d. We were sent for to visit Mr. A. M——, April

11th, 1846. Dr. Montague visited him, found him in a chill. The sinapisms, rubbing and Ether detailed in the above case, soon brought on a re-action. The pain in the head was very great, it would make him scream out aloud. Said "it was like some one was beating the top of his head off;" was restless; fever ran high. Soon after re-action came on, he commenced sweating, which continued through the exacerbation. He directed Sub. Murias Hyd. 10 grs., Pulv. Camphor, 2 grs., Pulv. Opii, ½ gr. to be given every 3 hours until the bowels were moved freely or 7 powders were given, and in case of no operation to be followed with a dose of Castor Oil. He also applied a blistering plaster to the back of the neck, and had cold applications applied to the head as often as the heat required them.

April 12th.—We visited the case together this morning, found him without much excitement, pain in the head severe; delirious; slight degree of opisthotonos, the contractions of the muscles of the back were not very rigid; pulse over 100; tongue covered with a white coat; eyes red and watery. The powders had operated without the oil; the blister had drawn well. We directed the powders to be commenced at 9 o'clock, and one to be given every 3 hours until he takes five, to be followed by Castor Oil if necessary. Liniment to be applied to the back, and when the fever rises, cold applications to the head; and the surface to be bathed frequently with warm vinegar and water.

Dr. Montague visited him at 4 o'clock P. M. He found him laboring under a high degree of excitement, his pulse was stronger then in the morning and somewhat hard; other symptoms as in the morning. He took 16 ounces of blood from the arm. He directed a powder to be given, when the excitement went down, of Quinine 8 grs., Precip. Carb. Ferri., 4 grs., Pulv. Dover 2 grs., every 3 hours during the remission.

13th—I visited Mr. M—— this morning at 8 o'clock, found him more rational; complains very much of the pain in his head back and hip of the right side; left eye some better, the right one worse, the cornea is completely opaque, and the pain in it is deep seated. His medicine had operated freely on the bowels—had to take the Castor Oil; had taken three of the Quinine powders. The muscles of the back are not contracted so much; when he is dozing he is talking incoherently, but stops when he is roused up. Continue the same treatment, that is, the Quinine powders every 2 hours until the fever increases, and Calomel, Camphor and Opium in the evening, 5 powders 3 hours apart, Oil after, if necessary; the Liniment to the back. And in addition, a blister over the affected hip, and a solution of Nitrate of Silver to the eye.

April 14th.—Visited him again, found an improvement in all the symptoms excepting the right eye and hip. The blister had drawn well; but there seemed to be a total paralysis of the limb. Continue the same treatment.

15th.—He appears still better, excitement last evening was less than before; rested some through the night; talked less; when he is raised up he has no disposition or ability to hold up his head; very little pain in the head. The eye and hip are no better. Continue the same treatment.

16th.—Still continues to improve, has some appetite, took some broth; thinks himself that he is better; his mouth is slightly affected by the Mercury; discontinue it; continue the tonic powders every 4 hours; directed the paralysed limb to be rubbed with Liniment, and increase of the irritation over the lumber vertrebra.

From this time there was a gradual improvement in Mr. M's. case, with the exception of the eye and hip. The eye never recovered. It appeared that the optic nerve was destroyed by the inflamation, as well as the opacity of the cornea that existed.

The leg improved so that he could get about with a crutch. He could move it, but had not strength enough in it to bear his whole weight upon it.

The nervous system received a shock that it never recovered from. His system was left a complete wreck. He died five months after of an attack of Billious Remittent fever contracted by exposure to night air.

The two cases detailed above are among those that contracted the disease repairing the farm and consequently occurred when the disease raged as an epidemic. The one given below was a saporadic case.

*Case* 3d. April 23d, 1849. I was called to see Mrs. J. who was taken sick the day before, with what they suppose to be the chill and fever. She was taken with a chill followed by a high fever and severe pain in the head, and this morning had another chill about the same time. The chill had passed off when I saw her. The pain in the head was very severe; she was tossing from one side of the bed to the other screaming aloud with the pain in the head, said "that her head would burst!" The pain extended down the back; the muscles of the back were contracted so as to form the trunk in a curve. The tonsils were slightly swelled; the tongue was covered with a white fur and moist; the pulse 120, and somewhat hard. All the secretions seemed to be suspended. As there were no symptoms present indicating the sinking of the vital energies of the system, I took 18 ounces of blood from the arm; directed a powder of Calomel 12 grs., Pulv. Opium ½ gr., Pulv. Camphor 2 grs., every 3 hours until 5 doses were taken, to be followed with Castor Oil if they do not operate; cold applications to the head; a blister to the back of the neck; Liniment to the throat and spinal column, below where the blister was applied; the surface to be sponged off frequently, with warm vinegar and water.

24th.—Visited Mrs. J. at 8 o'clock this morning, found her no better. There was a slight chillness about the same time this morning that she had had the chills before. The fever did not remit any; more stupor and delirium than yesterday. The medicine had operated freely on the bowels----had to take the Oil; blister drew well. Continue the powders, cold applications to the head and sponging the surface with warm vinegar and water.

4 o'clock P. M.----Very much the same; fever high, and some hardness of the pulse. I took 12 ounces of blood from the arm, and ordered a powder of Quinine 8 grs., Precip. Carb. Ferri 3 grs., Morphine $\frac{1}{8}$ gr. to be commenced 7 hours before the chill, if there was the slightest remission in the fever and given every three hours, and continued until the exacerbation next day.

25th, 8 o'clock in the morning.----The symptons very much as yesterday; no chillness this morning; a slight remission in the fever; had taken three of the Quinine powders, and I directed the fourth. Ordered the Calomel, Camphor and Opium in previous quantities to be commenced at 10 o'clock, and 4 powders to be given.

4 o'clock P. M. Found her with less fever than on yesterday; less delirium and opisthotonos. Directed the Quinine powders to be commenced at 12 o'clock to-night, and given every three hours until the exacerbation to-morrow.

26th. She is decidedly better this morning; less fever; no delirium; less pain in the head; and the opisthotonos entirely relieved. The Calomel operated without the Oil---- large quantities of dark, pitchy bile passed off from the bowels, of the consistence of tar. The throat is better. Continue the same treatment.

27th. She continues to improve with the exception of an erysipelatous inflammation of the throat under the chin, which commenced about the time the pain left the head. It is very painful, and appears to be spreading very rapidly; has no appetite----operations from the bowels are still dark and pitchy. Directed but two of the Calomel powders to-day. And the Quinine in 4 grains was combined with the Iron in 3 grain portions every 4 hours. And a strong solution of the Nitrate of Silver to the inflamed surface every 6 hours until it stops the inflammation.

28th. She still continues to improve; inflammation has spread in every direction since yesterday, but does not pain and burn so much as before the solution was applied; very black from the effects of the Nitarte of Silver. Some appetite to eat. Discontinued the Calomel powders. Continue Quinine powders, also the Nitrate of Silver solution to the inflamed surface.

29th. The inflammation has spread none since last visit; improving rapidly in every respect----appetite good. Discontinue all medicines excepting some to regulate the bowels.

31st. Still improving. Gave the case up as cured.

I could add other cases but this paper has swollen beyond the limits intended.

*Indian Point, Menard Co. Ill., May 26th,* 1851.

